# Qualitative Comparative Analysis and robust sufficiency

**DOI:** 10.1007/s11135-021-01157-z

**Published:** 2021-06-23

**Authors:** Michael Baumgartner

**Affiliations:** Department of Philosophy, University of Bergen, Postboks 7805, 5020 Bergen, Norway

**Keywords:** Qualitative Comparative Analysis (QCA), Solution types, Configurational comparative methods, Robust sufficiency, INUS causation

## Abstract

Some methodologists take the search target of Qualitative Comparative Analysis (QCA) to be causal INUS-conditions, others contend that QCA should instead be used to search for some form of sufficiency that is more substantive than mere Boolean sufficiency. While the notion of an INUS-condition has a long and uncontroversial definitional history, Adrian Dusa, in a recent paper, is the first to explicitly define a notion of substantive sufficiency, which he labels *robust sufficiency*. Dusa’s definition, however, is vacuous in real-life research contexts. As an alternative, the first part of this paper non-vacuously defines robust sufficiency and supplies a corresponding notion of minimality, which—I argue—captures Dusa’s conceptual intentions. In the second part, I then report and discuss the results of a series of simulation experiments benchmarking the performance of the different QCA solution types in recovering robust sufficiency and minimality.

**Supplementary Information:**

The online version contains supplementary material available at 10.1007/s11135-021-01157-z.

## Introduction

One topical anchor point of this special issue is the question which of QCA’s parsimonious, intermediate, or conservative solution types should be produced and interpreted primarily. The answer depends heavily on what the search target of QCA is taken to be. Different search targets are likely to be best captured by different solution types. In their introduction, Haesebrouck and Thomann (forthcoming, p. 8) take up and sharpen Thomann and Maggetti’s (2020) distinction between two approaches to QCA with different targets: the *SI-approach* “emphasizes the substantive interpretability of QCA results” (ibid. p. 363) and the *RF-approach* “emphasizes redundancy-free models” (p. 364). While both approaches agree that QCA searches for models that group exogenous factor values conjunctively and disjunctively, they disagree about the constraints imposed on the elements of these models. According to the SI-approach, each disjunct in a model, which itself can be a conjunction of factor values, must guarantee—in some yet to be specified sense—the occurrence of the outcome, even if that disjunct contains conjuncts that are not causes of the outcome. By contrast, the RF-approach demands that each disjunct in a model be exclusively composed of true causes of the outcome, even if the disjunct as a whole does not guarantee the outcome’s occurrence. Or put differently, the SI-approach does not mind false causal positives as long as the resulting models identify *substantively sufficient* conditions of the outcome, whereas the RF-approach wants to uncover as much as possible of the data-generating causal structure while avoiding false positives and, in return, does not mind if its models do not identify substantively sufficient conditions.

For the RF-approach, the implications that the choice of search target has for the choice of solution type are clear. The RF-approach defines causation in the vein of Mackie’s (1974) INUS theory, or modern variants thereof (e.g. Graßhoff and May 2001). The principal criterion INUS models have to satisfy is redundancy-freeness. In parsimonious solutions, redundancies are eliminated as much as is possible with QCA’s technical machinery, whereas the intermediate and conservative solution types often dispense with complete redundancy elimination. It follows that intermediate and conservative solutions are more likely than parsimonious ones to include causally irrelevant factor values and, hence, to commit false positives, meaning that the purposes of the RF-target are best served by the most parsimonious solution type (Baumgartner 2015).

It is much less clear what ramifications the search target of the SI-approach has for the preferability of solution types. The main reason is that the core notion on which the specification of the SI-target turns, *viz.* the notion of substantive sufficiency, has no definitional history. Although various representatives of the SI-approach explicitly say that QCA traces sufficiency and necessity relations that are “substantively interpretable” or more “meaningful” than mere Boolean dependencies (Schneider 2016, 2018; Thomann and Maggetti 2020), the only attempt at an explicit definition, to date, is due to Dusa (2019a), who labels his version of substantive sufficiency *robust sufficiency*: “[a] disjunct in a QCA solution is robustly sufficient if the outcome is guaranteed to occur in its presence” (p. 11). Plainly though, no conjunction of a handful of factor values—which is the maximal length of conjunctions in all QCA solution types—ever guarantees the occurrence of an outcome in our complex macroscopic world. Even the most commonplace of outcomes in the domain of social scientific QCA applications are influenced by a wide array of suitably interacting causes, not to mention causal background conditions enabling life on earth in the first place. A disturbance or interference anywhere in that array can prevent the occurrence of an outcome *Y* on any particular occasion, despite all causes featured in the best models of *Y* being co-instantiated. The Covid-19 pandemic painfully reminds us of this. Although, according to our best economic models, all causes of global economic growth were instantiated in January 2020 (IMF 2020), the pandemic, which no economic theory could possibly have incorporated, interfered and turned the growth rates into the deep negative. Correspondingly, no method in the social sciences (or in the special sciences, more generally) can seriously aim for robustly sufficient conditions *guaranteeing* the occurrence of outcomes. Hence, taken at face value, the search target of the SI-approach as defined by Dusa (2019a) is unattainable.

That means, in turn, that Dusa’s account of SI’s target cannot be taken at face value but most be modified. Before addressing the question what QCA solution type should be produced primarily by representatives of the SI-approach, its target must be defined in a non-vacuous manner. This is the topic of the first part of this paper. I render explicit in what sense, at best, sufficiency relations in QCA solutions can be expected to be robust and, thereby, to “guarantee” the occurrence of outcomes. Moreover, I introduce a notion of minimality for robustly sufficient conditions that captures Dusa’s idea that a substantive search target for the SI-approach should not be unnecessarily complex.

Against that backdrop, the second part then reports and discusses the results of a series of simulation experiments benchmarking the performance of the different QCA solution types in recovering the SI-target. I simulate a multitude of different data scenarios, including both crisp-set and fuzzy-set data, featuring fragmentation and noise of different degrees, and I measure the ratio of disjuncts in parsimonious, intermediate and conservative solutions as well as, for completeness, the ratio of sufficient truth table rows that satisfy, respectively, (non-vacuous) robust sufficiency and minimality. It turns out that the parsimonious solution is best suited for finding minimally robustly sufficient conditions, though at a modest overall success rate. When it comes to the discovery of (non-minimal) robust sufficiency, the truth table rows come out on top, with the conservative solution trailing closely behind. The paper’s supplementary material contains a detailed R script that allows for replicating all calculations and tests.

## Conceptual preliminaries

To level the field for readers adhering to different QCA approaches, I begin with some conceptual preliminaries required by my ensuing discussion.

I refer to the basic modeling devices of QCA as “factors” (many QCA methodologists prefer the label “conditions”). Factors are functions from (measured) properties into a range of values (typically numeric). They can be used to represent categorical properties that partition sets of units of observation (cases) either into two sets, in case of binary properties, or into more than two (but finitely many) sets, in case of multi-value properties. Factors representing binary properties can be *crisp-set* or *fuzzy-set*; the former can take on 0 and 1 as possible values, whereas the latter can take on any (continuous) values from the unit interval [0, 1]. Although factors processable by QCA can also represent multi-value properties, I will, for simplicity of exposition, develop my argument on the basis of crisp-set and fuzzy-set factors only.

As is common for QCA, I interpret values of a factor *X* as membership scores in the set of cases exhibiting the property represented by *X*. A case of type $X\!=\!1$ is a full member of that set, a case of type $X\!=\!0$ is a (full) non-member, and a case of type $X\!=\!\chi _i$, $0<\chi _i<1$, is a member to degree $\chi _i$. Since the explicit “Factor$=$value” notation yields convoluted syntactic expressions with increasing model complexity, I use a shorthand notation conventional in Boolean algebra (and QCA): membership in a set is expressed by italicized upper case and non-membership by italicized lower case Roman letters. Hence, I write “*X*” for $X\!=\!1$ and “*x*” for $X\!=\!0$. Moreover, I write “$\lnot X$” for the negation “not $X\!=\!1$”, “$X*Y$” for the conjunction “$X\!=\!1$ and $Y\!=\!1$”, “$X + Y$” for the disjunction “$X\!=\!1$ or $Y\!=\!1$”, “$X\rightarrow Y$” for the implication “if $X\!=\!1$, then $Y=\!1$”, and “$X \leftrightarrow Y$” for the equivalence “$X\!=\!1$ if, and only if, $Y\!=\!1$”.

Those Boolean operations constitute the heart of QCA’s formal machinery. In case of crisp-set factors, they are given a rendering in classical logic or set theory, which I do not reiterate here (for a canonical presentation see e.g. Lemmon 1965, ch. 1). Just the implication operator requires explicit introduction because the sufficiency relation, which is the topic of this paper, is defined on its basis. There are various equivalent ways of defining implication. In classical logic, “$X\rightarrow Y$” means that it is not the case that *X* is true and *Y* false or, equivalently, that *X* is false or *Y* is true; in set theory, implication is cashed out in terms of the subset operation: “$X\rightarrow Y$” means that *X* is a subset of *Y*.

For fuzzy-set factors, the classical Boolean operations must be translated into fuzzy logic. There exist numerous systems of fuzzy logic (for an overview cf. Hájek 1998), each of which comes with its own rendering of Boolean operations. In the context of QCA, the following fuzzy-logic renderings are standard: negation $\lnot X$ amounts to $1-X$, conjunction $X\!*\!Y$ to $\min (X,Y)$, disjunction $X+Y$ to $\max (X,Y)$, an implication $X\rightarrow Y$ is taken to express that the membership score in *X* is smaller or equal to *Y* (i.e. $X\le Y$), and an equivalence $X\leftrightarrow Y$ that the membership scores in *X* and *Y* are equal (i.e. $X=Y$).

Based on the implication operator, the notions of *sufficiency* and *necessity* are defined, which are the two Boolean dependence relations exploited by QCA:

*Sufficiency*: *X* is sufficient for *Y* iff $X\,\rightarrow \, Y$ (or equivalently: $x + Y$; and colloquially: “if *X* is present, then *Y* is present”);
*Necessity*: *X* is necessary for *Y* iff $Y\,\rightarrow \, X$ (or equivalently: $\lnot X\rightarrow \lnot Y$ or $y + X$; and colloquially: “if *Y* is present, then *X* is present”).

Importantly, claims of sufficiency and necessity carry no causal connotations whatsoever. They express mere association patterns or subset relations. While common QCA textbooks all define sufficiency and necessity in this way (see e.g. Schneider and Wagemann 2012, pp. 52–58; Dusa 2019b, pp. 125–126; Oana et al. 2021), representatives of QCA sometimes do not adhere to these definitions themselves, and erroneously suggest that sufficiency and necessity express more than mere association patterns. Ragin (2008, p. 53), for example, says “it is important to remember that the interpretation of any set-theoretic relation as either necessary or sufficient must be built on a solid foundation of theoretical and substantive knowledge” (or similarly Schneider 2016, pp. 782–783). In fact, no theoretical or substantive connection whatsoever is required for *X* to be sufficient or necessary for *Y*. If *X* is empty, *X* is sufficient for everything and everything is necessary for *X*; if *Y* is the universal set, everything is sufficient for *Y* and *Y* is necessary for everything. Plainly though, that sufficiency and necessity are not causally or theoretically loaded themselves does not preclude that more substantive relations can be defined on their basis. The INUS theory, for example, defines causation in terms of redundancy-free sufficiency and necessity structures. Or, the next section will define a notion of robust sufficiency that could serve as a worthwhile search target for the SI-approach.

The SI- and the RF-approach agree that a characteristic feature of QCA is that, unlike regression analytic methods, QCA does not focus on pairs of causes and outcomes aiming to quantify (net) effect sizes but that it attempts to group causes conjunctively, that is, into complex causes all elements of which need to be present in order for the outcome to occur, and disjunctively, that is, into alternative causes that can bring about the outcome independently of one another. Formally, models output by QCA are disjunctions of conjunctions, in disjunctive normal form,^1^ that are sufficient and necessary for the outcome.^2^ The following is an example (which I will come back to throughout this paper):

1 $$\begin{aligned} A\!*\!b \, + \, c\!*\!D \;\leftrightarrow \; E \end{aligned}$$

**Table 1:**
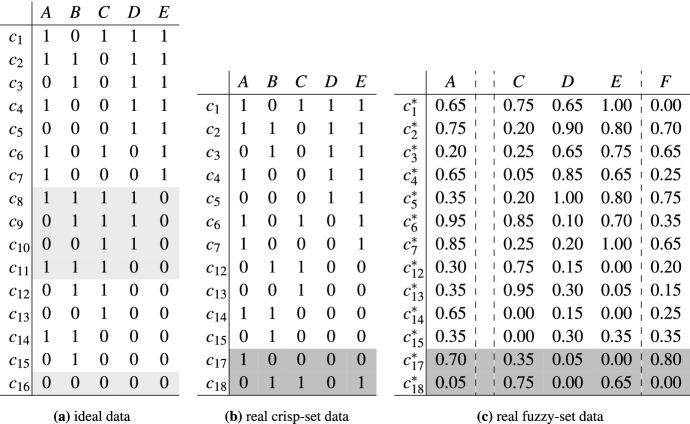
Ideal and real data generated from (1)

Light grey rows in (a) are missing from the real data in (b) and (c), dark grey rows in (b) and (c) are incompatible with (1). The relevant factor *B* is not included in (c); instead, the irrelevant *F* is included. The first column in each table provides case numbers

When causally interpreted (1) entails that $A\!=\!1$ and $B\!=\!0$ jointly cause $E\!=\!1$ on one path and that $C\!=\!0$ and $D\!=\!1$ constitute a complex cause of $E\!=\!1$ on another path. If this is the true causal structure regulating the behavior of the factors in (1), these factors can be co-instantiated in exactly the 16 configurations listed in Table 1a. Those are the configurations that are *compatible* with (1). Any other logically possibly configuration is *incompatible* with (1). For example, the configuration $A\!*\!b\!*\!C\!*\!D\!*\!e$, which results from case $c_1$ in Table 1a by switching *E* from 1 to 0, is incompatible with (1) because it features $A\!*\!b$ in combination with *e*, whereas (1) entails that whenever $A\!*\!b$ is given, so is *E*. Table 1a corresponds to ideal data on (1). If those data are processed by QCA, (1) is the only resulting model, that is, all solution types coincide (see the replication script for details).

However, real-life data are rarely ideal. They tend to be *fragmented*, meaning that not all configurations compatible with an investigated causal structure are actually observed. They are affected by *noise* or *measurement error* resulting in configurations incompatible with underlying data-generating structures. Or the set of analyzed factors may be *misspecified*, to the effect that relevant factors are not included in the data, while irrelevant factors are included. Table 1b and c illustrate real crisp-set and fuzzy-set data generated from structure (1), which is thus assumed to be the true *data-generating structure* behind those data. Configurations corresponding to the cases with light grey shading in Table 1a are missing in Table 1b and c. Instead, these latter tables feature configurations incompatible with (1) highlighted with dark grey shading. Moreover, the relevant factor *B* is missing from Table 1c and, instead, the irrelevant factor *F* is included.

In consequence, causal structures are often not correctly reflected by sufficiency and necessity relations in real-life data. For instance, in case $c_{17}$ of Table 1b, $A\!*\!b$ is given and *E* is not, meaning that $A\!*\!b$ is not strictly sufficient for *E*, contrary to what is entailed by (1). In case $c_{18}$, *E* is given without $A\!*\!b$ and $c\!*\!D$, meaning that their disjunction is not necessary for *E*, which also contradicts (1). Or, in $c^*_4$ of Table 1c, the membership score in $c\!*\!D$ ($\min (c,D)= 0.85$) is higher than the membership score in *E* (0.65), thus violating fuzzy-set sufficiency. That means, in order to infer a structure as (1) from real-life data by tracking sufficiency and necessity relations, those relations must not be interpreted in strict terms. Rather, suitable parameters of fit are needed to approximate strict sufficiency and necessity relations.

In QCA, those fit parameters are *consistency* and *coverage* (Ragin 2006). Consistency (*con*) and coverage (*cov*) of an implication $X\rightarrow Y$ in a data set with *n* cases (observations) are defined as follows:

$$\begin{aligned} con(X\rightarrow Y)= \frac{\sum \nolimits _{i=1}^n \min (X_i,Y_i)}{\sum \nolimits _{i=1}^n X_i}\quad cov(X\rightarrow Y)= \frac{\sum \nolimits _{i=1}^n \min (X_i,Y_i)}{\sum \nolimits _{i=1}^n Y_i} \end{aligned}$$

What counts as acceptable fit scores is defined in *thresholds* set by the analyst prior to the application of QCA. These thresholds determine how close a dependence in the data must approximate a strict crisp-set or fuzzy-set dependence in order to pass as one of sufficiency or necessity.

If QCA is applied for pure causal discovery purposes, as proposed by the RF-approach, setting the consistency threshold is a notoriously difficult task. On the one hand, slight changes in threshold settings may change models significantly, thus, making the threshold choice one of the most important steps of the analytic process. On the other hand, QCA faces a severe overfitting danger (Arel-Bundock 2019): if the threshold is set too high, even very mild noise levels may lead to overly complex parsimonious solutions—*viz.* the solutions of choice for causal discovery—that include causally irrelevant factor values.^3^ For example, if the consistency threshold is set to 0.865 when analyzing Table 1c, QCA returns the two parsimonious models (2) and (3), which erroneously ascribe causal relevance to *f*, *a*, and *F*.^4^

2 $$\begin{aligned} c\!*\!D\!*\!f \,+\, a\!*\!D\;&\rightarrow \; E \end{aligned}$$

3 $$\begin{aligned} c\!*\!D\!*\!f \,+\, a\!*\!F\;&\rightarrow \;E \end{aligned}$$

If the threshold is lowered to 0.85, only one model is returned and the overfitting disappears:

4 $$\begin{aligned} c\!*\!D\; \rightarrow \;E \end{aligned}$$

Although (4) does not capture the complete data-generating structure (1), it correctly captures one causal path and, beyond that, does not commit any fallacies. From the perspective of the RF-approach, (4) is the best model obtainable from Table 1c, because if the consistency threshold is lowered further, say, to 0.75, the model becomes too parsimonious, meaning that less true causal relations than could be inferred from the data are actually inferred:

5 $$\begin{aligned} D\; \rightarrow \;E \end{aligned}$$

That is, the problem of setting the consistency threshold in causal discovery with QCA is the problem of finding the sweet-spot between over- and underfitting in a context—unlike the current one—in which the true data-generating structure is not known.

On the face of it, threshold placement appears to be much less problematic if QCA is applied along the lines of the SI-approach. If the goal is not a fallacy-free inference to causation but an inference to some (yet to be specified) substantive form of sufficiency, threshold placement is not a means to finding a search target that is independent of that threshold, rather, it defines what degree of dependence the analyst is prepared to accept as one of sufficiency and, thereby, contributes to specifying what the target of the analysis is. All conditions found to comply with a chosen threshold are *ipso facto* sufficient for the outcome. Even though they are not causes, $c\!*\!D\!*\!f$ and $a\!*\!D$ are sufficient for *E* in Table 1c with consistencies of 0.85 and 0.82, respectively. Or, $A\!*\!b\!*\!C$ is strictly sufficient for *E* in Table 1b, from which QCA infers this conservative solution at a consistency threshold of 1:

6 $$\begin{aligned} c\!*\!D\; +\; A\!*\!b\!*\!C\;\rightarrow \; E \end{aligned}$$

If QCA models are not intended to exclusively contain true causes, the mere fact that they include causally irrelevant factor values—as *f* and *a* in (2) or *C* in (6)—does neither entail false positives nor that the models are overfitted. If (2) and (6) are interpreted in terms of some form of substantive sufficiency, they may well correctly reflect the intended search target, provided, of course, the sufficient conditions identified in these models not only meet the chosen consistency thresholds but are also substantive.

To determine whether the SI-approach is really less vulnerable to overfitting and threshold placement and to identify the solution type most suitable for its purposes, the next section turns to the obvious follow-up problem of defining a notion of sufficiency—robust sufficiency—substantive enough to be traced by QCA.

## Defining robust sufficiency

While Dusa (2019a) is not the first to suggest that the ultimate purpose of QCA might not be to facilitate fallacy-free inferences to causation but to some substantive form of sufficiency (see also e.g. Schneider and Wagemann 2012, p. 214; Schneider 2016, pp. 782–783), he is the first to acknowledge that this notion of substantive sufficiency requires explicit definition—just as does the notion of causation employed by the RF-approach. Such an explicit definition is needed because evaluating the performance of QCA is only possible if a precise grasp of its intended target—be it causation or something else—is available; and evaluating the performance of QCA, or any other method, is essential to understand under what discovery conditions its models are reliable and where its limitations are.

The following is Dusa’s (2019a, p. 11) definition of substantive sufficiency, which he labels *robust sufficiency*:^5^

[A] disjunct $\Phi$ in a QCA solution for an outcome *O* is robustly sufficient for *O* if and only if:

(i) $\Phi$ is sufficient for *O*.
(ii) No proper part of $\Phi$ is sufficient for *O*.
(iii) *O* is guaranteed to happen when $\Phi$ is present.

This definition has two severe shortcomings. First, it does not capture the conceptual intuition that Dusa describes in the surrounding text. He says he wants to define a notion of sufficiency that does not call for maximal redundancy elimination (i.e. maximal parsimony) in sufficient conditions, such that the intermediate and conservative solution types, which do not rigorously enforce redundancy-freeness, might regularly succeed in identifying robustly sufficient disjuncts. In fact, Dusa’s (2019a) main argumentative goal is to substantiate that the intermediate solution type is most successful at uncovering robust sufficiency. However, the notion of sufficiency appearing in conditions (i) and (ii) is the standard Boolean one. In consequence, (i) and (ii) simply amount to the ordinary definition of *minimal sufficiency* (cf. e.g. Baumgartner 2015, p. 844). It follows that only sufficient conditions that are rigorously freed of redundancies (i.e. that do not have sufficient proper parts) can satisfy (i) and (ii) and, thus, be robustly sufficient. As sufficient disjuncts in intermediate solutions are more likely to have sufficient proper parts than disjuncts in parsimonious solutions, it is clear prior to any benchmark experiments that parsimonious solutions are more successful than intermediate ones at recovering robust sufficiency as defined above. Hence, when taken literally, Dusa’s definition merely selects a proper subset of disjuncts in models of the parsimonious solution type, *viz.* those that, on top of satisfying redundancy-freeness, also satisfy condition (iii). That is not Dusa’s intention, nor the intention of representatives of the SI-approach more generally.

The second shortcoming is that condition (iii) turns on the notion of “guaranteeing” an outcome to happen, which remains obscure throughout Dusa’s article. In the passage following the definition of robust sufficiency, he says that “Boolean minimization guarantees the outcome will always occur” (Dusa 2019a, p. 11). It is beyond me in what sense of “guarantee” the occurrence of an outcome *in the world* could possibly be guaranteed—or even influenced—by Boolean minimization, which, after all, is a syntactic operation performed *on a model*. Moreover, as anticipated in Sect. 1, if condition (iii) is meant to require that *O* is ensured to occur (without exception) whenever $\Phi$ is true, the whole definition of robust sufficiency becomes vacuous in real-life discovery contexts. The reason is simply that disjuncts in QCA models of all solution types comprise a maximum of 5–6 factor values, yet no outcome in our complex macroscopic world can be determined by a handful of factor values only. In consequence, condition (iii) is unsatisfiable in other than artificial toy examples.

To get a precise understanding of the search target of the SI-approach, I have to define a workable notion of substantive sufficiency myself. To this end, the remainder of this section attempts to capture what Dusa might have in mind by rectifying the shortcomings of his definition. For conceptual clarity, I disentangle the ideas behind conditions (i) and (ii), on the one hand, and condition (iii), on the other. I will define, first, a non-vacuous notion of robust sufficiency that recovers the idea behind condition (iii), and second, a notion of minimality that corresponds to conditions (i) and (ii), but without calling for redundancy-freeness in standard Boolean sufficiency relations.

As a concrete background for this discussion, consider the following empirical interpretation of the causal structure (1). Suppose a city has two power stations: a hydroelectric and a nuclear plant. Let *A* express that the hydroelectric plant is operational and *D* that the nuclear plant is operational and let *b* and *c* express the absence of defects in the power lines connecting the two plants to the city’s electricity grid. Hence, $A\!*\!b$ is one cause of the city being power supplied (*E*) and $c\!*\!D$ is another. Even though these are two causes of *E*, they can neither separately nor jointly guarantee that the city is power supplied because neither $A\!*\!b$ nor $c\!*\!D$ nor $A\!*\!b\!*\!c\!*\!D$ are strictly sufficient for *E* under all possible circumstances, *viz.* in all real-life data sets on the factors in structure (1). As illustrated in Table 1, real-life data typically feature cases such that $A\!*\!b$ or $c\!*\!D$ are given while *E* is not, or cases whose minimum membership scores in $A\!*\!b$ or $c\!*\!D$ are higher than their membership scores in *E*. Since not even the true causes of *E* guarantee the occurrence of their outcome, the same holds *a fortiori* for disjuncts in QCA models inferred from real-life data on structure (1). In fact, when such models are built with consistency thresholds below 1 their disjuncts may not even be strictly sufficient for *E* in the data from which they have been inferred. The fuzzy-set data in Table 1c, for example, do not contain any strictly sufficient conditions for *E* whatsoever.

$A\!*\!b$ and $c\!*\!D$ cannot guarantee *E* because they only reflect a small subset of all causes influencing a real city’s power supply, the majority of which remaining latent. Two types of latent causes must be distinguished: *enablers* and *off-path causes*. Enablers are causes that must be instantiated in the background in order for the (non-latent) measured causes to be causally effective, and off-path causes are causes that can bring about an outcome independently of the measured ones, that is, via causal paths that do not go through the measured causes. For example, $A\!*\!b$ and $c\!*\!D$ only effectuate the city’s power supply provided that there is no power outage, the plants’ personnel is not on strike, or the city has a demand for power in the first place. Hence, the absence of outages and strikes and the presence of a power demand are enablers of $A\!*\!b$ and $c\!*\!D$. By contrast, electricity import is an example of an off-path cause of *E*, as it can power supply the city independently of $A\!*\!b$ and $c\!*\!D$, that is, on causal paths not including the factors in structure (1).

Uncontrolled variation in enablers and off-path causes tends to give rise to data confounding and noise, which, in turn, prohibits strict Boolean dependencies from becoming manifest in data. Still, if there is no power outage, no strike, and a sufficient power demand while electricity is not being imported etc., the city can only be power supplied on causal paths through the plants and their power lines. In other words, if all enablers are instantiated and all off-path causes are suppressed, the only factors that can make a difference to *E* are the ones influencing *E* on paths through the factors in structure (1). In such an idealized context, nothing can interfere with the causal influence of $A\!*\!b$ and $c\!*\!D$ on *E* and nothing can trigger *E* independently of these causes, meaning that all variation in *E* must be accountable for by variation in factors *A*, *B*, *C*, or *D*. Idealized contexts are free of data confounding and noise. They allow for generating *ideal data*—which notion will be essential for my account of robust sufficiency:

* Ideal data*: An ideal data set $\delta ^{id}$ over a set ${\mathbf {F}}$ of analyzed factors comprising an outcome *O* and exogenous factors ${\mathbf {F}}\setminus \{O\}$ is a set that includes all and only those configurations of the factors in ${\mathbf {F}}$ that are empirically possible in a context in which all enablers of ${\mathbf {F}}\setminus \{O\}$ are instantiated and all off-path causes of *O* are suppressed.

What counts as an empirically possible configuration is determined by the causal structure, if any, regulating the behavior of the factors in ${\mathbf {F}}$. A configuration is empirically possible if, and only if, it is compatible with that structure when enablers are instantiated and off-path causes suppressed. As we have seen in the previous section, if the set of analyzed factors is $\{A,B,C,D,E\}$ and (1) the causal structure, the 16 configurations in Table 1a (and no others) are empirically possible. If, by contrast, there is no causal structure regulating the behavior of these factors (i.e. if they are causally independent), all 32 logically possible configuration of these factors are empirically possible. An ideal data set may contain multiple cases instantiating empirically possible configurations, as long as it contains at least one instance of every empirically possible configuration and no instances of empirically impossible configurations, that is, no configuration incompatible with the underlying causal structure.

Importantly, while strict Boolean dependencies may be wanting in real-life data such that no conditions guarantee the occurrence of the outcome, ideal data over a set of factors whose behavior is regulated by some deterministic causal structure always feature strict Boolean dependencies.^6^ That is, even though $A\!*\!b$, $c\!*\!D$ or $A\!*\!b\!*\!c\!*\!D$ are not strictly sufficient for *E* in many real-life data sets, they are strictly sufficient for *E* in ideal data over the set $\{A,B,C,D,E\}$—as is illustrated in Table 1a. And there exist many more conditions, apart from deterministic causes, that are strictly sufficient for outcomes in ideal data. In Table 1a, for example, all configurations $c_1$ to $c_7$ are strictly sufficient for *E*. Correspondingly, while conditions inferred by QCA from data over a set of factors ${\mathbf {F}}$ cannot be expected to be strictly sufficient for an outcome *O* in all data sets over ${\mathbf {F}}$, they can reasonably be expected to be strictly sufficient for *O* in ideal data over ${\mathbf {F}}$. It might therefore be a worthwhile target for QCA to search for conditions that, although not guaranteeing *O* in all contexts, can be said to guarantee *O* in an idealized context in which enablers are instantiated and off-path causes suppressed. That means a condition $\Phi$ could be defined to be robustly sufficient for *O* if, and only if, $\Phi$ is strictly sufficient for *O* in ideal data over ${\mathbf {F}}$.

This simple definition, however, is prone to trivialization. Sufficient conditions in QCA research have the logical form of conjunctions. But conjunctions can be strictly sufficient for outcomes in data—both ideal and not ideal—for trivial logical or conceptual reasons. For example, a logical contradiction as $\,X\!*\!x\,$ is strictly sufficient for every outcome *O* in every data set (ideal or not), no matter the underlying causal structure. Similarly, every conjunction containing *O*, e.g. $X\!*\!y\!*\!O$, is strictly sufficient for *O* in every data set. Or, a conjunction may be strictly sufficient for *O* because there is a conceptual dependence between that conjunction and *O*.^7^ Of course, trivial sufficiency relations as $\,X\!*\!x\rightarrow O\,$ or $\,X\!*\!y\!*\!O \rightarrow O\,$ are not scientifically meaningful. They should, therefore, not count as cases of robust sufficiency. Hence, turning the simple definition into one that provides a notion of robust sufficiency worthwhile to be traced in scientific studies requires provisions safeguarding against trivialization.

One approach to avoid trivialization—the one adopted by Dusa (2019a, p. 11)—is to make the notion of robust sufficiency apply to disjuncts in models of QCA solution types only. As disjuncts in QCA models can neither comprise logical contradictions nor the outcome and as QCA must only be applied to factor sets free of conceptual dependencies, QCA (properly conducted) does not issue trivial sufficiency relations, which, according to that approach, disqualifies them as cases of robust sufficiency. But if being a disjunct in a QCA model is necessary for being robustly sufficient, it becomes impossible for mere definitional reasons that there exist robustly sufficient conditions that QCA fails to find. Robust sufficiency then cannot figure as search target for QCA that is conceptually independent of the method itself. To avoid that consequence, I prefer to provide a general definition of robust sufficiency that is not restricted to disjuncts in QCA models. This, in turn, calls for explicit definitional restrictions disqualifying trivial sufficiency relations from passing as instances of robust sufficiency: robustly sufficient conditions must be non-contradictory, conceptually independent of the outcome, and may not include the outcome as a conjunct.

Combining all the above considerations yields the following definition of robust sufficiency:

*Robust sufficiency (RS)*: A conjunction of one or more factor values $\Phi$ is robustly sufficient for an outcome *O* relative to a set of factors ${\mathbf {F}}$ containing *O* if, and only if, (a) $\Phi$ is not logically contradictory, (b) $\Phi$ does not contain *O* as a conjunct, (c) $\Phi$ and *O* are conceptually independent, and (d) $\Phi$ is strictly sufficient for *O* in ideal data $\delta ^{id}$ over ${\mathbf {F}}$.

For convenience, I will refer to conditions that are robustly sufficient in the sense defined by (RS) as *RS-conditions* of their outcomes. Before moving on to recover the ideas behind Dusa’s (2019a, p. 11) conditions (i) and (ii), four important characteristics of definition (RS) must be highlighted. First, robust sufficiency as defined in (RS) is not vacuous. $A\!*\!b$ qualifies as robustly sufficient for *E* despite the fact that $A\!*\!b$ does not guarantee *E*, say, in case $c_{17}$ of Table 1b. The reason is that some enabler must be latently varying in $c_{17}$. If the background is properly idealized such that enablers are constantly instantiated, as in Table 1a, $A\!*\!b$ is strictly sufficient for *E*. Since that sufficiency relation is moreover non-trivial, $A\!*\!b$ passes as an RS-condition of *E*. What matters for robust sufficiency as defined in (RS) is not—as in Dusa’s (2019a) definition—sufficiency *simpliciter* but only sufficiency in ideal data. By that standard, a host of further RS-conditions of *E* can be built from the factors in Table 1, for example, $c \!*\!D$, $b\!*\!c \!*\!D$, $A\!*\!b \!*\!d$, $A\!*\!B\!*\!c \!*\!D$, or $A\!*\!b\!*\!c \!*\!d$, etc.

Second, what counts as an RS-condition is *relative* to the set of analyzed factors. A conjunction may be an RS-condition for an outcome relative to a set ${\mathbf {F}}_1$ but not relative to another set ${\mathbf {F}}_2$. To illustrate, while $A\!*\!b$ in structure (1) is robustly sufficient for *E* relative to the set ${\mathbf {F}}_1=\{A,B,C,D,E\}$, it is not robustly sufficient for *E* relative to the set ${\mathbf {F}}_2=\{A,B,C,D,E,S\}$ that results from ${\mathbf {F}}_1$ by adding factor *S* representing strikes of plant personnel. To see this, note that the absence of strikes is a precondition for the plant’s operationality and its faultless connection to the electricty grid to cause the city’s power supply. That is, $A\!*\!b$ is strictly sufficient for *E* in ideal data over ${\mathbf {F}}_1$ because the absence of *S*, *viz.* *s*, is an enabler that is constantly instantiated in the idealized background of such data. But since *S* is included in ${\mathbf {F}}_2$, it is no longer an enabler fixed to a particular value but allowed to vary in ideal data over ${\mathbf {F}}_2$. In consequence, ideal data over ${\mathbf {F}}_2$ contain cases such that $A\!*\!b$ is combined with striking personal *S* (i.e. $A\!*\!b\!*\!S)$, in which cases the city is not power supplied. It follows that $A\!*\!b$ is not strictly sufficient for *E* in ideal data over ${\mathbf {F}}_2$ and thus not an RS-condition of *E* relative to ${\mathbf {F}}_2$.

Third, many conditions that are sufficient for an outcome in the analyzed data do not pass as RS-conditions. To see this, take the parsimonious QCA solution inferred from the non-ideal data in Table 1b over the set ${\mathbf {F}}_1$ (at a consistency threshold of 1):

7 $$\begin{aligned} D \, + \, A\!*\!C \;\leftrightarrow \; E \end{aligned}$$

That model has an overall consistency of 1 and coverage of 0.75. Both disjuncts are strictly sufficient for *E* in Table 1b. However, neither of them is robustly sufficient for *E* in ${\mathbf {F}}_1$ because neither of them is strictly sufficient for *E* in the corresponding ideal data in Table 1a. Those ideal data contain various cases such that *D* or $A\!*\!C$ are given while *E* is not, *viz.* cases $c_8$ to $c_{11}$. These empirically possible cases just happen to be unobserved in the fragmented non-ideal data of Table 1b. Hence, just as real-life data can be misleading with respect to causal dependencies, they can also be misleading with respect to robust sufficiency.

Finally, fourth, RS-conditions are not the same as causes. Causes *explain* outcomes. They are composed of difference-makers of outcomes, meaning that for each component *X* of a cause there exist cases such that a change in *X* is associated with a change in the outcome when everything else stays the same. That does not hold for components of RS-conditions. In our leading example of structure (1), $A\!*\!b\!*\!d$, for instance, is an RS-condition of *E* but the non-operationality of the nuclear plant, *d*, obviously does not cause *E*. Still, it holds that whenever $A\!*\!b\!*\!d$ is given in ideal data, *E* is given as well. That is, although RS-conditions may be composed of causally irrelevant factor values that do not explain an outcome, they provide a *recipe* for bringing about the outcome within an ideal causal background. Despite the fact that they do not guarantee the outcome in all circumstances, they can be said to guarantee the outcome in ideal circumstances—which is the only sort of “guarantee” models of empirical processes can reasonably be expected to deliver. Although an RS-condition $\Phi$ does not supply a fail-safe recipe for producing the corresponding outcome *O*, knowing that $\Phi$ is an RS-condition may be valuable even when realizing $\Phi$ under non-ideal circumstances fails to trigger *O*. Such knowledge tells us, first, that there must exist alternative circumstances in which $\Phi$ is actually associated with the desired outcome and, second, where to search the culprit for the failed attempt to produce *O*, *viz.* in non-instantiated enablers. In that light, knowledge of RS-conditions optimizes the process of finding suitable interventions that purposefully generate a desired outcome.

Overall, definition (RS) approximates the conceptual intuition that Dusa (2019a, p. 11) hints at in condition (iii) as much as is possible without falling into the vacuity pitfall. (RS) provides a relative notion of robust sufficiency that is not restricted to disjuncts in QCA models. It is neither vacuous nor trivially satisfiable, and knowledge of RS-conditions is valuable for purposefully bringing about an outcome. It follows that RS-conditions can be argued to constitute a worthwhile search target for the SI-approach of QCA.

Still, RS-conditions cannot be the only target of the SI-approach. If they were, there would be no incentive for eliminating redundancies at all. As a conjunction $X_1\!*\!\ldots \!*\!X_n$ returns the minimum $\min (X_1,\ldots , X_n)$, it follows that the more conjuncts it contains, the higher the chances that $\min (X_1,\ldots , X_n)$ does not exceed the membership scores that the cases in data $\delta ^{id}$ have in the outcome *O* and, if compliance with conditions (a) to (c) of (RS) is ensured, the higher the chances that $X_1\!*\!\ldots \!*\!X_n$ is robustly sufficient for *O*. The conjunctions of factor values with the highest number of conjuncts that are (non-trivially) sufficient for the outcome are the unminimized configurations of the truth table that meet a chosen consistency threshold in the analyzed data.^8^ That means, if QCA’s search target were RS-conditions per se, the models with the highest likelihood of successfully recovering that target would simply consist in disjunctive concatenations of the sufficient truth table rows. Although Collier (2014, p. 124) has indeed suggested that QCA analysts should dispense with algorithmically processing truth tables and, rather, concentrate on the truth table itself, that position is not mainstream among representatives of the SI-approach. According to the mainstream position, the preferred QCA solution type is the intermediate one (Haesebrouck and Thomann forthcoming, p. 9), in which some factor values that are redundant to preserve sufficiency are eliminated.

Indeed, the advantages of RS-conditions without redundant elements are obvious. If the goal is to purposefully produce an outcome, less complex recipes are clearly preferable over more complex ones, for they are easier and less expensive to implement. As we have seen above, Dusa’s (2019a, p. 11) attempt at capturing that intuition in conditions (i) and (ii) collapses onto Boolean minimality. By contrast, the separate rendering of the idea behind condition (iii) in definition (RS) now allows us to impose a minimality constraint on RS-conditions that does not reduce to Boolean minimality.

*Minimality (M)*: A robustly sufficient condition $\Phi$ for an outcome *O* relative to a set of factors ${\mathbf {F}}$ is minimal if, and only if, $\Phi$ does not have a proper part $\Phi '$ that is robustly sufficient for *O* relative to ${\mathbf {F}}$, where a proper part $\Phi '$ is the result of eliminating one or more conjuncts from $\Phi$.

The notion of sufficiency appearing in the definiens of (M) is *robust* sufficiency as defined in (RS) and not sufficiency simpliciter as in (i) and (ii). In consequence, (M) does not impose redundancy-freeness on sufficient conditions but on RS-conditions. An RS-condition being minimal means that none of its proper parts is robustly sufficient for the outcome. For convenience, I shall henceforth refer to minimally robustly sufficient conditions, that is, to conditions satisfying both (RS) and (M), as *MRS-conditions* of their outcomes.

To illustrate, the following is the intermediate QCA solution for Table 1c over the set ${\mathbf {F}}_3 =\{A,C,D,E,F\}$ using a consistency threshold of 0.85 and directional expectations correctly mirroring the causal relevancies in structure (1) from which that Table was simulated (cf. the replication script for details):

8 $$\begin{aligned} A\!*\!c\!*\!D\, +\, c\!*\!D\!*\!F\; \rightarrow \; E \end{aligned}$$

The disjuncts of this model reach consistency scores of 0.89 and 0.9 in Table 1c, yet they are both strictly sufficient for *E* in ideal data over ${\mathbf {F}}_3$. As these sufficiency relations are not trivial, both disjuncts are RS-conditions of *E*. However, neither of them is an MRS-condition, as both of them contain a proper part that is itself an RS-condition of *E*, *viz.* $c\!*\!D$, which is strictly sufficient for *E* in ideal data over ${\mathbf {F}}_3$. In other words, all that is needed to purposefully produce *E* under ideal circumstances is $c\!*\!D$. Additionally instantiating *A* or *F* is redundant.^9^

In sum, I submit that RS- and MRS-conditions constitute transparent targets for the SI-approach that do not suffer from the shortcomings of Dusa’s (2019a, p. 11) proposal. Learning about RS-conditions from QCA amounts to learning how to purposefully produce an outcome of interest under ideal circumstances, and MRS-conditions give us a maximally cost-effective recipe for that purpose. The immediate follow-up question now is which QCA solution type(s) most reliably uncover(s) RS- and MRS-conditions. The next section addresses that question by means of benchmark experiments.

## Benchmarking

To quantify the success rates of the different QCA solution types in discovering RS- and MRS-conditions, we need to move beyond particular examples and benchmark the solutions’ performance in a variety of discovery circumstances. To this end, I set up a series of inverse search trials, first, randomly drawing data-generating structures (or ground truths), second, simulating different types of data from those structures, and third, inferring the different QCA solution types to measure the ratios of RS- and MRS-conditions they contain. This section first explains the details of the test setups and benchmark criteria and then presents the test results.

To get a statistically significant performance assessment, I draw 1000 data-generating structures $\Delta$ from the factor set ${\mathbf {F}}_\Delta =\{A,B,C,D,E,F\}$ where *D* is designated to be the outcome. Each $\Delta$ has between 2 and 9 causally relevant factor values distributed over 1–3 causal paths, such that in some structures many factors in ${\mathbf {F}}_\Delta$ are causally irrelevant to *D*, while in other structures all factors are relevant. In a first benchmark experiment, I then simulate crisp-set data with different fragmentation and noise levels from these structures while randomly varying the sample sizes, and I analogously simulate fuzzy-set data in a second experiment. There are slight differences between these experiments, both in data simulation and analysis. I introduce the main simulation and analysis principles against the backdrop of the crisp-set trials and point out, along the way, how the fuzzy-set trials differ.

I begin by producing an ideal data set $\delta ^{id}$ for every $\Delta$, comprising one case per configuration of the factors in ${\mathbf {F}}_\Delta$ compatible with $\Delta$, which yields 1000 ideal data sets over ${\mathbf {F}}_\Delta$ each containing 32 cases.^10^ To investigate how different degrees of fragmentation (i.e. limited diversity) affect QCA’s performance, I then simulate three degrees of fragmentation—low, medium, high—by removing 2, 9, and 16 of the cases, randomly drawn (without replacement), from each of the ideal data sets $\delta ^{id}$. This results in 1000 data sets of each of the following types: $\delta ^{6}$, $\delta ^{28}$, and $\delta ^{50}$ with $6.25\%$, $28.125\%$, and $50\%$ fragmentation, respectively. The degree of fragmentation can be thought of as the ratio of empirically possible configurations that remain unobserved in a study. Next, the sample sizes of these fragmented data are varied by expanding them by copies of randomly selected cases contained in them.^11^ More specifically, for each data set $\delta ^x_i$ a number *k*, such that $10\le k\le 100$, is drawn and *k* cases contained in $\delta ^x_i$ are sampled, with replacement and equal selection probability, and added to $\delta ^x_i$. As a result, the sample sizes of $\delta ^{6}$, $\delta ^{28}$, and $\delta ^{50}$ now vary freely between 26 and 130 cases. Importantly, all of these data sets only contain cases compatible with their underlying data generating structure $\Delta$, meaning they are entirely noise-free.

Next, I introduce noise. In the crisp-set experiment, two noisy data sets are created from every noise-free $\delta ^{6}$, $\delta ^{28}$, and $\delta ^{50}$ by replacing, respectively, 5% and 15% of the cases compatible with $\Delta$ by randomly drawn cases incompatible with $\Delta$—which incompatibilities can be thought of as resulting from measurement error or confounding. Each case compatible with $\Delta$ has equal probability of being replaced by an incompatible one and each incompatible case has equal probability of being drawn, meaning that noise is introduced without bias. In the fuzzy-set experiment, noise is introduced by fuzzifying $\delta ^{6}$, $\delta ^{28}$, and $\delta ^{50}$. This is done in two ways. In the first, I add a number drawn from the sequence (0, 0.05, 0.1, 0.15, 0.2) to every 0 in $\delta ^{6}$, $\delta ^{28}$, and $\delta ^{50}$ and subtract such a number from every 1, yielding low fuzzification. In the second, I draw the numbers to add and subtract from the sequence $(0,0.05,0.1,\ldots ,0.45,0.55, 0.6)$, which produces high fuzzification. Overall, this data simulation results in the following 15 data types comprising 1000 data sets each, with randomly varying sample sizes, where superscripts indicate the degrees of fragmentation, that is, the percentages of unobserved empirically possible configurations, and subscripts the noise levels, that is, the percentages of cases incompatible with $\Delta$ in case of crisp-set data and the degrees of fuzzification in case of fuzzy-set data:

**Table:** 

	Crisp-set:	Fuzzy-set:
Low fragmentation:	$\delta ^{6}_{0},\; \delta ^{6}_{5},\; \delta ^{6}_{15}$	$\delta ^{6}_{\text {low}},\; \delta ^{6}_{\text {high}}$
Medium fragmentation:	$\delta ^{28}_{0},\; \delta ^{28}_{5},\; \delta ^{28}_{15}$	$\delta ^{28}_{\text {low}},\; \delta ^{28}_{\text {high}}$
High fragmentation:	$\delta ^{50}_{0},\; \delta ^{50}_{5},\; \delta ^{50}_{15}$	$\delta ^{50}_{\text {low}},\; \delta ^{50}_{\text {high}}$

These 15,000 data sets are then analyzed by QCA, as implemented in the R package **QCA** (Dusa 2021), with three different consistency thresholds $con^\theta$, yielding $9\times 3 = 27$ test types in the crisp-set experiment and $6\times 3 =18$ test types in the fuzzy-set experiment. In the crisp-set experiment, I set $con^\theta$ to 1, 0.8, and 0.75, respectively. In the fuzzy-set experiment, I choose thresholds of 0.9, 0.8, and 0.75, because consistencies of 1 are rare in fuzzy-set data.^12^ Each of the resulting test types consists of 1000 data sets that are analyzed by QCA. In each analysis, I build the conservative solution type (CS), the parsimonious type (PS), and two variants of intermediate solution types, best intermediate (ISb) and default intermediate (ISd). In ISb, I assume (non-conjunctural) directional expectations that correspond to the true causal relevancies in $\Delta$, and in ISd, directional expectations are set to each exogenous factor in ${\mathbf {F}}_\Delta$ being positively relevant to the outcome. For completeness, I additionally build a solution type labelled TT that simply consists in a disjunction of the truth table rows that meet the chosen $con^\theta$.

I measure the quality of these solution types by determining the ratio of disjuncts in each (non-empty) model that are RS- and MRS-conditions, respectively. As all of the conditions tested for that purpose are parts of QCA models, which do not contain trivial sufficiency relations by default, compliance with the trivialization provisions (a) to (c) in definition (RS) can be assumed as given and does not need to be checked. This greatly facilitates testing whether (RS) is satisfied: a disjunct $\Phi$ in a QCA model passes as an RS-condition of the designated outcome *D* relative to ${\mathbf {F}}_\Delta$ if, and only if, $\Phi$ is strictly sufficient for *D* in the corresponding ideal data $\delta ^{id}$ over ${\mathbf {F}}_\Delta$. This test yields an *RS-ratio*, that is, a ratio of the number of RS-conditions in a QCA model to the number of disjuncts in that model, for every QCA model. To determine whether a disjunct $\Phi$ in a QCA model is an MRS-condition, I build all proper parts $\Phi '$ of $\Phi$ and check for all of these parts whether they are RS-conditions relative to ${\mathbf {F}}_\Delta$. If that check is positive for at least one $\Phi '$, $\Phi$ is not an MRS-condition.^13^ Or contrapositively put, $\Phi$ is an MRS-condition if, and only if, that check is negative for all of its parts $\Phi '$. This test yields the *MRS-ratio*, that is, the ratio of the number of MRS-conditions in a QCA model to the number of disjuncts in that model, for every model.

**Fig. 1:**
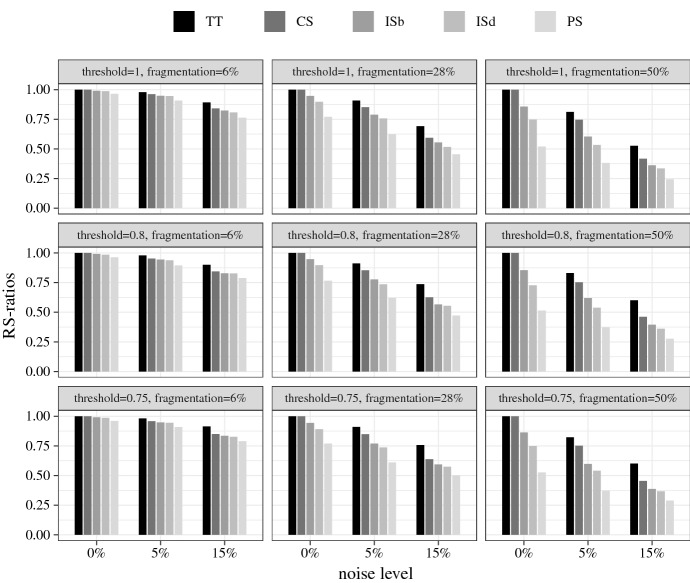
RS-ratios for the crisp-set experiment. Each ratio is an average over all models recovered in all 1000 trials in a corresponding test type. “TT” stands for sufficient truth table rows, “CS” for conservative, “ISb” for best intermediate, “ISd” for default intermediate, and “PS” for parsimonious solution

**Fig. 2:**
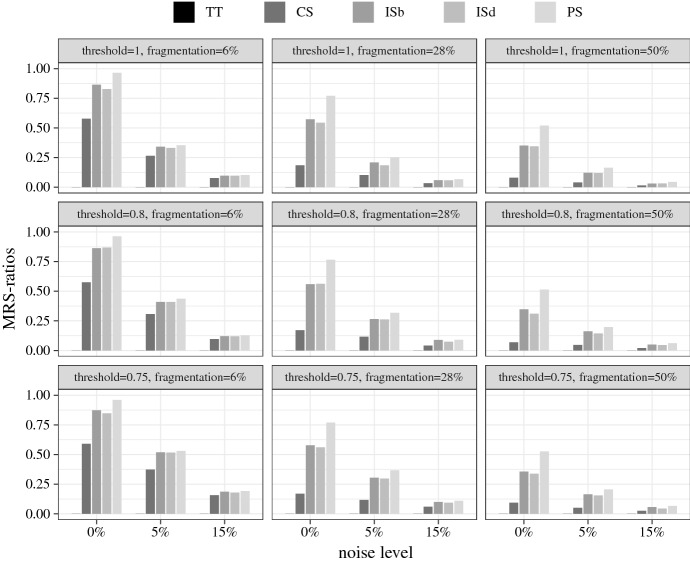
MRS-ratios for the crisp-set experiment

**Fig. 3:**
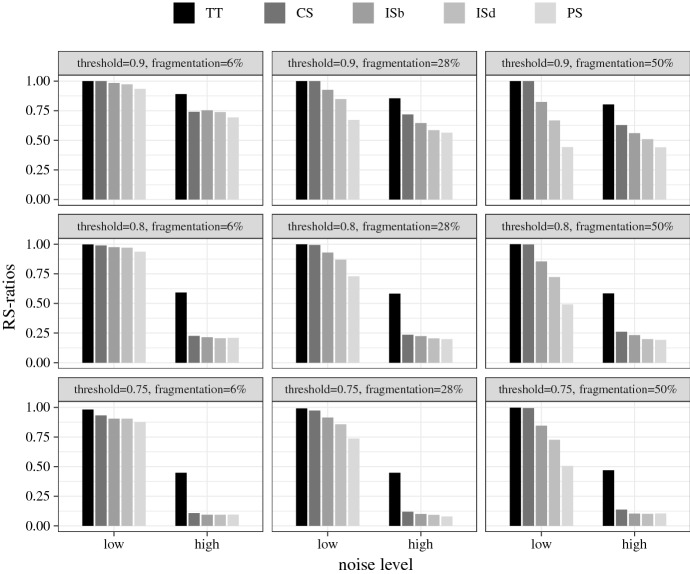
RS-ratios for the fuzzy-set experiment

**Fig. 4:**
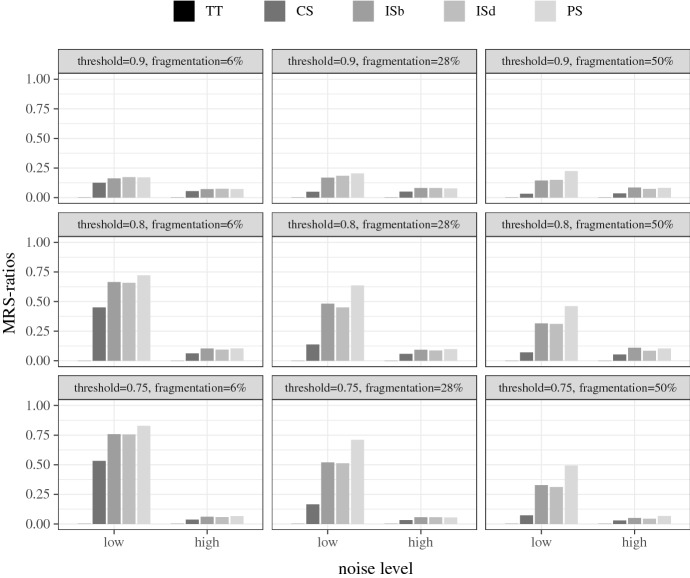
MRS-ratios for the fuzzy-set experiment

Average RS- and MRS-ratios are the results I report. Figures 1 and 2 plot the RS- and MRS-ratios for the crisp-set experiment, broken down by the different test types, and Figs. 3 and 4 the corresponding ratios for the fuzzy-set experiment. The bars represent the ratios averaged over all models recovered in all 1000 analyses performed in each test type. Null results (i.e. empty solutions), which regularly occur, for example, when there are no configurations in the data that meet $con^\theta$, are not counted in these scores, that is, QCA is not punished for abstaining from drawing an inference when the data are too noisy.^14^ The reported ratios, hence, express the average share of (M)RS-conditions contained in all non-empty QCA solutions of a particular test type. In other words, they determine how successful the different solution types are at recovering (M)RS-conditions under the discovery circumstances of a test type, provided the analysis is not abandoned due to the impossibility to meet $con^\theta$. To illustrate, the value 1.00 represented by the two leftmost bars in the top-left panel of Fig. 1 means that in all 1000 analyses of data of type $\delta ^{6}_{0}$ at $con^\theta = 1$, in which that consistency threshold can be reached, all sufficient truth table rows and all disjuncts in all models of the conservative QCA solution type are RS-conditions. Or the value 0.07 displayed by the right-most bar in the bottom-right panel of Fig. 2 means that 7% of disjuncts of all models in non-empty parsimonious solutions inferred from the 1000 data sets of type $\delta ^{50}_{15}$ at $con^\theta = 0.75$ are MRS-conditions.

The first and most noticeable finding is that, although I identified MRS-conditions as more substantive search target, QCA *de facto* is much more successful at finding RS-conditions than MRS-conditions. Averaged over all test types, the solution most successful at recovering RS-conditions (unsurprisingly) is TT with overall RS-ratios of 0.88 in the crisp-set experiment and 0.81 in the fuzzy-set experiment, whereas the solution most successful at recovering MRS-conditions, averaged over all test types, is PS with an overall MRS-ratio of 0.39 in the crisp-set and 0.29 in the fuzzy-set experiment.

These low overall MRS-ratios need to be differentiated further. There are significant differences in MRS-ratios between different noise levels and degrees of fragmentation. When inferred from crisp-set data without any noise, 75% of disjuncts in PS models, averaged over all degrees of fragmentation and threshold settings, are MRS-conditions, as compared to 60% in ISb, 58% in ISd, 28% in CS, and 0% in TT models. When crisp-set data feature only mild noise levels, however, those scores plummet. Meager 20% of disjuncts in PS models are MRS-conditions in the trials with non-zero noise—and this is still the best performance of all solution types. QCA’s success at MRS-discovery is also very sensitive to degrees of fragmentation. In data with low fragmentation, 51% of disjuncts in PS models, averaged over all noise levels and threshold settings, are MRS-conditions, as compared to 48% of ISb, 47% of ISd, 34% of CS, and 0% of TT models. By contrast, in data with medium and high fragmentation, those scores are cut in half. On the upside, the MRS-ratios in the crisp-set experiment are only mildly affected by the placement of $con^\theta$. When analyzing data with non-zero noise, setting $con^\theta$ to 0.75 improves the MRS-ratios for all solution types by roughly 7 percentage points compared to $con^\theta =1$. Hence, when QCA is used for MRS-discovery from crisp-set data the overfitting danger is less severe than in case of causal discovery (see Sect. 2 above). Still, these poor overall success rates show that QCA should only be used for MRS-discovery when the data are very clean, in which case the parsimonious solution type performs best.

As there are no trials with completely noise-free data in the fuzzy-set experiment (which are the trials that boost the crisp-set scores), the MRS-ratios averaged over all trials in that experiment are significantly lower than the crisp-set scores. With an overall MRS-ratio of 0.29, the parsimonious solution type again comes out on top. And there is a further parallel to the crisp-set trials: noise has a massive negative effect on the performance of all solution types. When averaged over all degrees of fragmentation and threshold settings, $50\%$ of disjuncts in PS models are MRS-conditions in the trials on low noise data. In the high noise trials that score collapses to $8\%$. Contrary to the crisp-set experiment, however, threshold placement is very consequential for the MRS-ratios in the fuzzy-set trials. If noise levels are low, lowering $con^\theta$ from 0.9 to 0.75 triples the MRS-ratios of all solution types. That means, there is a substantive overfitting danger when MRS-conditions are inferred from fuzzy-set data.

While PS has an edge in MRS-discovery over the other solution types at a modest success rate, TT outperforms the other solution types in discovering RS-conditions in both experiments at a solid success rate. Averaged over all trials in the crisp-set experiment, TT reaches an RS-ratio of 0.88, with the other solution types trailing with scores between 0.83 (CS) and 0.63 (PS). As the fuzzy-set experiment does not comprise trials on entirely noise-free data, these overall scores are again lower in that experiment, but the scoreboard looks similar, with the only difference that TT, with its overall RS-ratio of 0.81, has a clear margin over the other solution types. CS, which is the next best solution type, has an overall RS-ratio of 0.67. As in case of MRS-discovery, noise and fragmentation hamper QCA’s success at finding RS-conditions, but while even the best performing solution type becomes unreliable in finding MRS-conditions with increasing data deficiencies, the best performing solution type, TT, maintains a solid RS-ratio even when analyzing highly fragmented and noisy data. Finally, there is a notable difference between the two experiments when it comes to the placement of the consistency threshold. In the crisp-set experiment, lowering $con^\theta$ from 1 to 0.75 is associated with a slight increase in RS-ratio of an average of about 2 percentage points in all solution types. By contrast, in the fuzzy-set experiment, all solution types score significantly better when $con^\theta$ is set higher. TT, for example, has an overall average RS-ratio of 0.72 at $con^\theta =0.75$, which increases to 0.92 at $con^\theta =0.9$, or the RS-ratio of CS jumps from 0.54 at $con^\theta =0.75$ to 0.85 at $con^\theta =0.9$. That means, while the consistency threshold should not be maximized when tracing MRS-conditions, especially in case of fuzzy-set data, QCA more reliably finds RS-conditions in fuzzy-set data with a higher consistency threshold.

Let us take stock. QCA is an unreliable tool for finding MRS-conditions when the data are affected by high fragmentation or high noise. Only if fragmentation is not high and noise is low, the parsimonious solution type may be used for MRS-discovery. In contexts of no more than 28% fragmentation and low noise, 71% of disjuncts in PS models, averaged over both experiments, are MRS-conditions when the consistency threshold is set to 0.75.

By contrast, QCA is a reliable tool for finding RS-conditions even when the data feature high fragmentation and noise, especially when the sufficient truth table rows are reported. Averaged over all trials in both experiments, 85% of all disjuncts in the non-standard solution type TT amount to RS-condition. Of course, apart from being a non-standard solution type, sufficient truth table rows contain redundancies in every single trial of the test series, meaning the recipes they provide are never cost-effective. But the standard conservative solution CS, which removes some redundancies, still performs acceptably well in RS-discovery averaged over all trials in both experiments: 75% of its disjuncts are RS-conditions some of which even amount to cost-effective recipes, especially in low fragmentation and noise scenarios. If the placement of the consistency threshold is moreover optimized in both experiments, the overall performance of CS can be increased to 84% of disjuncts, on average, being RS-conditions.

## Discussion

The results of these benchmarking experiments run counter to standard tenets of the SI-approach, most notably its distinctive preference for the intermediate solution type, which seems unjustified in the light of my results. If SI’s search target are robustly sufficient conditions, TT or CS should be the solutions of choice, whereas if minimally robustly sufficient conditions are targeted, the focus should be on PS and on ensuring very high data quality. Representatives of the SI-approach can refute my analysis on two grounds: (I), they can accept (RS) and (M) as valid definitions of QCA’s search targets but reject my benchmarking of these relations, or (II), they can reject that (RS) and (M) faithfully capture what they have in mind by a substantive non-Boolean notion of sufficiency. In the remainder of the paper, I address these possible objections in turn.

One way to raise objection (I) might be to point out that Dusa (2019a) claims to have shown the superiority of intermediate solutions for the discovery of robustly sufficient conditions in a test series and to conclude from this that there must be something wrong with my benchmarking. Unfortunately, I am unable to determine how exactly Dusa’s benchmarking relates to mine because, for the reasons given in Sect. 3, his explicit definition of robust sufficiency, when taken literally, requires rigorous redundancy-freeness in Boolean sufficiency relations, which unquestionably is more frequently achieved in parsimonious solutions than in intermediate ones. In order to nonetheless have intermediate solutions come out on top, he must be benchmarking something else than robust sufficiency as he defines the notion. However, it does not become clear what exactly he is testing instead. Apart from these ambiguities, Dusa’s test series is limited to one single data-generating structure and he follows Baumgartner and Thiem (2020) in simulating noise-free crisp-set data only. He merely tests QCA’s performance in analyzing data generated by one specific causal structure (*viz.* the same one used by Baumgartner and Thiem 2020) when the only type of data deficiency is fragmentation (limited diversity). Hence, even if it should turn out that his tests are faithful to his definition of robust sufficiency, the fact that intermediate solutions come out on top in such a limited test setting, provides hardly any justification for an overall preferability of the intermediate solution type.

By contrast, I test a notion of minimal robust sufficiency that does not require redundancy-freeness in Boolean sufficiency relations, and I cash out Dusa’s “guaranteeing” intuition in a manner that is satisfiable in real-life discovery contexts. Against that refined conceptual background, I simulate data from a multitude of randomly drawn data-generating structures, some of which are simple, some of which are complex, such that some simulated data sets comprise causally irrelevant factors and some do not. I consider both crisp-set and fuzzy-set data, which are not only affected by fragmentation but also by various degrees of noise. My data have varying sample sizes, I analyze them with three different consistency thresholds, and I give QCA the benefit of the doubt by (unrealistically) supplying directional expectations that match the true causal structure perfectly. Given the generality of this test setup, I submit that my benchmarking provides a more solid basis for generalizing the results than Dusa’s—and one of those results is that researchers interested in (minimal) robust sufficiency should not focus on intermediate solutions.

Another criticism in the vein of (I) might be to reject RS- and MRS-ratios as fair benchmark measures—which, to repeat, feature the number of a model’s (M)RS-conditions in the numerator and its total number of disjuncts in the denominator. A version of this criticism was advanced by an anonymous reviewer of this paper who alleges that these ratios are biased against IS because PS models tend to have fewer disjuncts than IS models, meaning their (M)RS-ratios have lower denominators and are, hence, higher on average. This objection neglects that (M)RS-ratios can be increased not only by lower denominators but also by higher numerators, and models with more disjuncts have higher chances of hitting the target and thus receiving higher numerators. That PS models have fewer disjuncts means that they are more cautious in ascribing modeled properties (e.g. minimal robust sufficiency) to conditions than other, more audacious solution types. (M)RS-ratios are neither biased towards cautiousness nor audaciousness. They reward cautiousness if that leads to avoiding false positives just as they reward audaciousness if that leads to hitting the target more frequently. Correspondingly, since minimality as defined in (M) is very difficult to infer from fragmented or noisy data, the cautiousness of PS is the modeling strategy receiving the highest overall MRS-ratios, whereas the audaciousness of TT, which typically contains far more disjuncts than IS or PS, is rewarded by the highest overall RS-ratios because the inference to robust sufficiency as defined in (RS) is much less demanding, even if data have various deficiencies.

Put bluntly, (M)RS-ratios are the ratios of hits among shots fired. They measure the accuracy of a solution type in recovering the intended target. Averages of these ratios over large enough samples can be seen as estimates of the probability that a particular disjunct in a model is an (M)RS-condition. I submit that this is exactly what researchers targeting (M)RS-conditions are looking for. They want to find a recipe for an outcome of interest. The overall RS-ratio of a solution type tells them how probable it is that a disjunct in a model of that type guarantees the occurrence of the outcome under ideal circumstances, while the overall MRS-ratio is an estimate of the probability that such a recipe is cost-effective. That said, it shall not be denied that there may be other measures viably benchmarking the performance of QCA solution types by quantifying different properties of that performance. Such alternative measures may yield different scoreboards. Hence, replacing (M)RS-ratios by alternative benchmark measures is a live option for representatives of the SI-approach wanting to refute my analysis along the lines of objection (I). Making that objection compelling would, however, require defining and justifying such alternative measures and establishing in a test series that IS performs most successfully in RS- or MRS-discovery when success is quantified on the basis of these measures.

Even if (M)RS-ratios are accepted as most pertinent benchmark measures, my test series can be criticized for falling short of covering all conceivable discovery contexts. Most importantly, I only simulate randomly distributed noise. But, of course, noise may be biased in real-life data; for example, certain types of measurement error may be more frequent than others. Also, the frequency of configurations compatible with a ground truth is unbiased in my test series, which may likewise be violated in real-life data. And I do not benchmark QCA’s performance in analyzing multi-value data. In light of these limitations, I have not conclusively shown that the intermediate solution should never be the solution of choice when tracking (minimal) robust sufficiency as defined in (RS) and (M). Hence, representatives of the SI-approach may defend the intermediate solution type by presenting concrete discovery contexts in which IS outperforms the other types.

As far as objection (II) is concerned, it goes without saying that there are multiple conceivable ways to define a notion of sufficiency that has more substance than the mere subset relation expressed in Boolean sufficiency. My proposal of Sect. 3 attempts to do justice to two constraints. First, I want to stay as close as possible to Dusa’s (2019a) suggestion that substantive sufficiency should in some sense “guarantee” the outcome without comprising redundant elements. Second, I want my notion of substantive sufficiency to be traceable, in principle, by the technical machinery currently employed in QCA. I am confident that any alternative definition that does justice to these two constraints has to be so close to my proposal that my benchmark tests will not yield relevantly different results. But an alternative account of substantive sufficiency may, of course, abstain from doing justice to these constraints.

It could, for instance, be contended that substantive sufficiency must be underwritten by *counterfactual dependence*. One such option might be to stipulate that $\Phi$ counts as substantively sufficient for an outcome *Y* if, and only if, there exist circumstances ${\mathscr {F}}_1$ in which both $\Phi$ and *Y* occur, such that had $\Phi$ not occurred in ${\mathscr {F}}_1$, *Y* would not have occurred either, as well as circumstances ${\mathscr {F}}_2$ in which neither $\Phi$ and *Y* occur, such that had $\Phi$ occurred in ${\mathscr {F}}_2$, *Y* would have occurred also. Such a proposal would approximate the notion of substantive sufficiency to causation as defined, for example, by Lewis (2000) or Woodward (2003) (cf. also Haesebrouck and Thomann forthcoming, pp. 20–22). While no doubt conceptually (and philosophically) interesting, there is currently no theory available connecting the technical machinery of QCA and its solution formulas, which are both firmly embedded in classical logic and set-theory, to relations of counterfactual dependence, which violate core axioms of classical logic, for example, the principle of extensionality.^15^ That is not to say that such a theory could not be developed, but in its absence the merits of defining substantive sufficiency and, hence, the search target of the SI-approach on the basis of counterfactual dependence cannot be assessed, not to mention benchmarked.

Alternatively, it could be suggested that substantive sufficiency has nothing to do with “guaranteeing” the outcome but, rather, with the non-trivialness of the sufficiency relation. For example, Schneider (2016, pp. 785–787) suggests that a meaningful sufficient condition is one that meets the chosen consistency and PRI thresholds, whereas a meaningful necessary condition has to meet the consistency and RON thresholds. PRI and RON are measures of the degrees to which conditions are, respectively, sufficient and necessary for both the positive and negative outcomes; low PRI and RON scores are interpreted as indicating that corresponding sufficiency and necessity relations are trivial. Although Schneider does not make these suggestions in the context of discussing QCA’s search targets, it could straightforwardly be exported to that context by proposing that the search target of the SI-approach consists in sufficient and necessary conditions that not only meet consistency but also PRI and RON thresholds. That, of course, would be a clearly defined target and the question which QCA solution type most successfully recovers that target could easily be answered in a simulation study. Hence, if non-trivialness indeed is their intended search target, I would invite representatives of the SI-approach to conduct such a study.

On a more general note, even if proponents of the SI-approach remain unconvinced by my analysis and reject (RS) and (M) for not capturing their intentions, I hope that they can at least agree with me—and with Dusa (2019a), for that matter—that their search target urgently needs to be explicitly defined in a non-ambiguous and non-vacuous manner. The criticism of SI’s preferred solution type, raised mainly by representatives of the RF-approach, cannot be effectively countered by alluding to some undefined notion of sufficiency that is more substantive than mere Boolean sufficiency. To firmly establish intermediate solutions as QCA’s preferable solution type, a precise understanding is needed of what it is exactly that intermediate solutions are best equipped to tell us about a modeled system. As it stands, the only way to characterize what an intermediate solution is is by alluding to the algorithm that generates them: an intermediate solution is the solution that results from Boolean minimization based on a user-defined set of directional expectations. But that, obviously, cannot be the solution’s purpose. Without a clear account of the solution’s purpose, which must be independent of the algorithm that generates it, intermediate solutions cannot be benchmarked, as it is impossible to determine whether they serve their purpose or not. And a solution type whose performance cannot be benchmarked is futile because it is indeterminate how reliable it is under different discovery circumstances.

In comparison, the RF-approach can characterize exactly what the purpose of its preferred solution type, the parsimonious one, is, *viz.* to identify causal INUS-conditions as defined by the INUS theory or modern variants thereof. Such a characterization is entirely independent of the algorithm that generates parsimonious solutions and, consequently, the success rate of the parsimonious solution can be precisely quantified and tested. In light of the results of this paper, one might consider to include the identification of MRS-conditions among the purposes of the parsimonious solution. But even though it is the most successful solution in MRS-discovery, in particular, at low consistency thresholds and when data quality is very high, I take QCA’s overall performance in finding MRS-conditions to be too low to extend the purpose of the parsimonious solution.

By contrast, based on my results I can recommend the use of sufficient truth table rows or of the conservative solution for finding (non-minimal) robustly sufficient conditions as defined in (RS), even when data are affected by high fragmentation and noise. Although robustly sufficient conditions with a host of redundant elements are not a search target that is equally substantive as MRS- or INUS-conditions, (RS) still furnishes a precise account of what exactly we can learn from truth table rows and conservative solutions, respectively—an account that is independent of the algorithms generating those solutions and, thus, allows for benchmarking.

## Supplementary Information

Below is the link to the electronic supplementary material.

Supplementary material 1 (R 19 KB)

Supplementary material 2 (R 74 KB)

## Data Availability

Data are made available in supplementary R scripts. Replication code is made available in supplementary R scripts.

## Footnotes

1 An expression is in disjunctive normal form iff it is a disjunction of one or more conjunctions of one or more literals (i.e. factor values; Lemmon 1965, p. 190).

2 Contrary to the RF-approach, the SI-approach may accept a disjunctive normal form as a model even if it is not necessary for the outcome. That difference is irrelevant for my argument in this paper.

3 That holds for data featuring both fragmentation and noise, but, as Baumgartner and Thiem (2020) have shown, not for data only featuring fragmentation. Parsimonious models inferred from noise-free data do not contain false positives.

4 Models (2) to (5) only have an implication arrow “$\rightarrow$” as main operator because their coverage scores are too low, i.e. between 0.42 and 0.62 (see the replication script for details).

5 I change Dusa’s bullet points to Roman numbers to facilitate referencing. Moreover, note that this definition is a very recent proposal and not an established tenet of the SI-approach.

6 According to the standard interpretation of quantum mechanics, processes among fundamental particles may be inherently indeterministic (e.g. Albert 1992). Hence, it shall not be presumed here that all causation is deterministic. Strict Boolean dependencies are only guaranteed to exist in ideal data over *deterministic* causal structures, which are the types of structures common in the macroscopic world to which QCA is typically applied.

7 For example, the conjunction of being a woman and having a child is strictly sufficient for being a mother in all data sets (ideal or not).

8 A truth table is an intermediate calculation device for QCA that simply indicates for every minterm (i.e. conjunction of all exogenous factors) instantiated in the data whether or not it meets the consistency threshold (see e.g. Schneider and Wagemann 2012, ch. 4).

9 Note that although the MRS-condition $c\!*\!D$ is also a cause of *E* in this example, MRS-conditions are not always causes, as is famously illustrated in the so-called *Manchester Factory Hooters* example (Mackie 1974, pp. 81–87).

10 ${\mathbf {F}}_\Delta$ has one endogenous and five exogenous factors. All ideal data sets with five exogenous factors representing binary properties whose behavior is regulated by a (non-empty) causal structure feature $2^5=32$ configurations.

11 Just as fragmentation and noise, the sample size affects the overall performance of QCA. Due to computational limitations, I can only systematically vary two of these three data properties in a controlled manner. Hence, the effect of the third property, which I determined to be sample size, must be randomized away.

12 According to standards of good practice of the SI-approach, consistency thresholds should be set based on individual properties of the resulting truth tables and not mechanically as done here. I have not seen an argument convincing me that such individualized threshold placement produces more reliable models overall, but do not have the space to delve into this issue here. Suffice it to say that the practice is followed very rarely in actual QCA studies, where conventional thresholds of 0.8 or 0.75 are mostly chosen mechanically. I recognize that readers who are convinced that individualized threshold placement leads to more reliable models overall will view my experiments as simulating data analysis of low quality. But even so, my results will expose general tendencies in how threshold placement affects QCA’s performance overall.

13 To illustrate, take the first disjunct in (8): $A\!*\!c\!*\!D$. These are its proper parts: $A\!*\!c$, $A\!*\!D$, $c\!*\!D$, *A*, *c*, and *D*. $A\!*\!c\!*\!D$ is not an MRS-condition because one of its parts, *viz.* $c\!*\!D$, is itself and RS-condition.

14 Dusa (2019a, pp. 12–13) seems to suggest that null results should be counted as incorrect. I decidedly disagree. Abstaining from drawing an inference if fit thresholds cannot be met is the only correct handling of overly noisy data.

15 In very brief, a classical sufficiency claim as “$A\rightarrow B$” is truth-functional, meaning its truth value is determined by the truth values of *A* and *B*. By contrast, a counterfactual conditional as $A \,\Box\!\!\rightarrow B$ is not truth-functional; its overall truth value is not determined by the truth values of *A* and *B*. “Had the Titanic not hit the iceberg, it would not have sunken”, where both antecedent and consequent are false, is commonly considered a true counterfactual. By contrast, “Had the Titanic not hit the iceberg, it would have warped into a spaceship”, where again both antecedent and consequent are false, is commonly considered a false counterfactual. Reasoning with counterfactual claims requires a sophisticated theoretical apparatus that goes way beyond classical logic or set theory (for a seminal treatment see Lewis 1973).
